# Examining the influence of the pedagogical beliefs on the learning management system usage among university lecturers in the Kurdistan Region of Iraq

**DOI:** 10.1016/j.heliyon.2022.e09687

**Published:** 2022-06-06

**Authors:** Twana Tahseen Sulaiman, Anuar Shah Bali Mahomed, Azmawani Abd Rahman, Mazlan Hassan

**Affiliations:** a Department of Business and Management, School of Management and Economics, University of Kurdistan Hewlêr, Erbil, Kurdistan Region, Iraq; b School of Business and Economics, Universiti Putra Malaysia, 43400, UPM Serdang, Selangor Darul Ehsan, Malaysia

**Keywords:** Learning management system, Pedagogical beliefs, Constructivist pedagogical beliefs, Traditional pedagogical beliefs, TAM

## Abstract

The pedagogy method academicians in the Kurdistan Region follow has not been reformed since 1992. There is a need to test different pedagogical beliefs, including constructivist and traditional pedagogical beliefs. This study examined the effect of pedagogical beliefs on technology adoption among university lecturers. The present study extends the Technology Acceptance Model (TAM) by including lecturers' pedagogical beliefs and examining them in Arab counties. A questionnaire was distributed during the COVID-19 pandemic, and 393 data were collected from university lecturers. The study examined how the constructivist and traditional pedagogical beliefs influence the model's three constructs: perceived ease of use, perceived usefulness, and actual use. This study has new finding that contributes to TAM, and the reasons are deliberated.

## 1 Introduction

The COVID-19 pandemic causes deleterious effects on lecturers working within academic settings. Fear of technology and inadequate awareness of software created ongoing challenges for lecturers as well. Furthermore, one must also consider that the lecturers’ pedagogical beliefs were suffering grief and change resulting from using a learning management system (LMS). LMS is an application that provides an extensive set of tools for faculty members to administrate the learning process outside and inside the lecture hall [1]. In this platform, some vital facilities such as wiki, chat, forum, downloading and uploading documents, e-portfolio, and teamwork have been employed [2]. Most learning management system platforms have remarkable features such as quizzes, online video tutorials, plagiarism checking, interim assessments, and group discussions [3]. Moodle LMS is provided beneficial in an organisational setting, with many educational institutions are taking the opportunity to increase the usage of Moodle LMS [4].

The pedagogy method academicians in the Kurdistan Region follow has not been reformed since 1992 [5]. The Ministry of higher education and scientific research of the Kurdistan Region (MHESR) struggled to reform the old pedagogy to the most suitable pedagogical method for the students in 21 century [6]. The government of the Kurdistan Region invests much money in providing LMS tools for universities, but the utilisation is still low among academicians [7, 8]. It has been emphasised by many studies that LMS tools have a significant impact on modifying the teaching method [9]. Therefore, adopting and using LMS may lead to transforming the old pedagogy into the modern pedagogy method. However, the Ministry of higher education desires to convert the pedagogical method from a teacher-centred pedagogy to a learner-centred pedagogy [10]. It has been stated by Ibrahim and Nat [11] that this pedagogical change can be done through academician empowerment through ICT integration in all characteristics of teaching. According to Tondeur [9], the best way to change the pedagogical teaching method is to adopt ICT within teaching inside the classroom. Hence, the Ministry is committed to implementing new educational technologies into academic activity.

Lecturers are supposed to change perception as the COVID-19 has taken its course. If perceptions have been affected by the pandemic, this may significantly contribute to the education context. Also, it included the change in the perceptions of how they believe toward using LMS. This quick move to online learning prompted lecturers to transfer from traditional teaching to online teaching directly. During the pandemic, lack of preparedness, virtual experience, and technical problems fostered virtual learning perception as inferior performance to face-to-face education.

There is reliable testimony that effective LMS use needs changes in pedagogical beliefs that develop confidence for ongoing learning and adaptability to change [2]. Such an approach forces lecturers to question their pedagogical beliefs and recognize what they still need to learn and what kind of lecturers they are (and want to be) in terms of a lifelong learning perspective. Lecturers must do their best to transform “traditional” classroom teaching into practical activities. Consequently, it seems essential to provide lecturers with technical and pedagogical knowledge that allows them to take advantage of new technologies by exploiting LMS features that make them unique.

The correlation between lecturers' pedagogical beliefs and their technology usage has been examined comprehensively by scholars such as Ertmer, Sang and Tondeur. However, this relationship remains unclear and needs more empirical research [12]. Given the uniqueness of lecturers’ pedagogical beliefs and the lack of a clear understanding of this relationship, the aim of this quantitative study is to investigate this relationship further in the era of the COVID-19 pandemic.

This current study contributes to filling the research gap in Arabic online learning literature. First, it examined constructivist and traditional pedagogical beliefs as factors influencing LMS usage, which studying this relationship is limited. Previous studies have suggested investigating factors that affect LMS usage. This paper presents significant perceptions of how constructivist and traditional pedagogical beliefs affect LMS usage. Nevertheless, prior studies have shown the influences of pedagogical beliefs on LMS adoption independently across various circumstances. However, pedagogical factors determined in this study are rarely integrated into a study to examine the actual use of educational technology within the online platform. The current research advances the understanding of the combined effects of constructivist and traditional pedagogical beliefs on LMS usage. Moreover, this paper has extended prior literature on the TAM model to examine LMS usage by adding constructivist and traditional pedagogical beliefs as external factors to the original TAM model. Those factors play a critical role in clarifying how actual technology uses are manipulated within the virtual learning environment.

The remainder of this paper is organised as follows. The following section provides a literature review in section 2, a theoretical framework, and sets out the study's hypotheses in section 3. Then, the research methodology is presented in Section 4. Section 5 provides the results, followed by the findings in section 6 and a discussion in section 7. The conclusion is set out in the final section.

## 2 Literature review

Pedagogical beliefs are defined as the beliefs and attitudes that lectures hold relating to the philosophy of teaching and how teaching should be proceed out [13]. Many researchers agree that lecturers' pedagogical beliefs influence how technology is used and integrated within the classroom [14]. It can be predicted that there is a significant relationship between pedagogical beliefs and Moodle LMS usage. There are two types of pedagogical beliefs as traditional and constructivist pedagogical beliefs. Lecturers holding the traditional beliefs tend to consider teaching a method for transferring knowledge to the students [9]. To achieve this, they prefer to control the classroom as well as students' performance and the educational content. They act as the authority to determine the correctness of students’ learning outcomes. The students are treated as passive recipients of confirmed knowledge [15]. Windschitl and Sahl [16] found that the impact of technology usage on educators who have traditional pedagogical beliefs was minimal. These findings appear to confirm that traditional pedagogical beliefs would be an obstacle to effective technology utilisation among lecturers.

Moreover, Tondeur, van Braak [14] found that lecturers with traditional pedagogical beliefs did not significantly impact their attitudes toward using (ICT) tools in their classroom. Tondeur, van Braak [14] defined teachers with constructivist pedagogical beliefs. Lecturers motivated by constructivist teaching pedagogy would see lecturing as a process of facilitating students’ creativity in an understanding of the phenomena they experience [17]. These lecturers organise the learning environment to encourage active understanding among the students, and they are responsive rather than prescriptive in determining what and how to learn.

It has been predicted by many researchers that constructivist pedagogical beliefs affect computer usage among faculty [18]. Gyamfi [13] states that instead of many technical, organisational factors affecting technology adoption and usage, institutional factors like lecturers' pedagogical beliefs influence utilising LMS by lecturers. However, Tondeur [9] emphasises that different types of pedagogical approaches of a particular lecturer may conflict with utilising new educational technology. So far, there has been no study investigating the effects of pedagogical beliefs on the LMS in the higher education context in the Kurdistan Region of Iraq. Hence, it is essential to pay attention to the lecturer's pedagogical beliefs on LMS use in the Kurdistan Region.

Previous studies have extensively observed the relationship between pedagogical beliefs and technology usage among teachers [14, 18, 19, 20]. It has been indicated that pedagogical beliefs have a significant role in choosing the technology system to align with their teaching strategy. Ertmer, Ottenbreit-Leftwich [19] and Judson [21] suggest that lecturers with student-centred beliefs tend to be more active technology users. Tondeur, Hermans [22] observe that lectures with constructivist beliefs are using technology.

On the other side, Becker [23] argued that lectures with teacher-centred beliefs tend to use technology in their classroom but might not use it in a more student-centred style. A study of faculty members from Greece realized a relationship between pedagogical beliefs and integrating LMS in teaching processes. They found that lecturers' pedagogical beliefs are a significant motivator for LMS and give many disciplines to faculty members [24]. The need to better align lecturers' preparation for the integration of technology with pedagogical issues has been noted by many researchers recently [9]. A study from Greece found a positive relation between pedagogical beliefs and integrating and using LMS by lecturers. Likewise, instructors' s teaching beliefs give faculty members many disciplines [24]. Research on educational technology suggests that the utilisation of ICT tools in general and LMS, in particular, can only be fully comprehended when lecturers’ pedagogical beliefs are credited [14, 25, 26]. More specifically, lecturers choose specific LMS platforms to integrate into their classroom practices [14].

## 3 Technology acceptance model (TAM)

**Figure 1:**
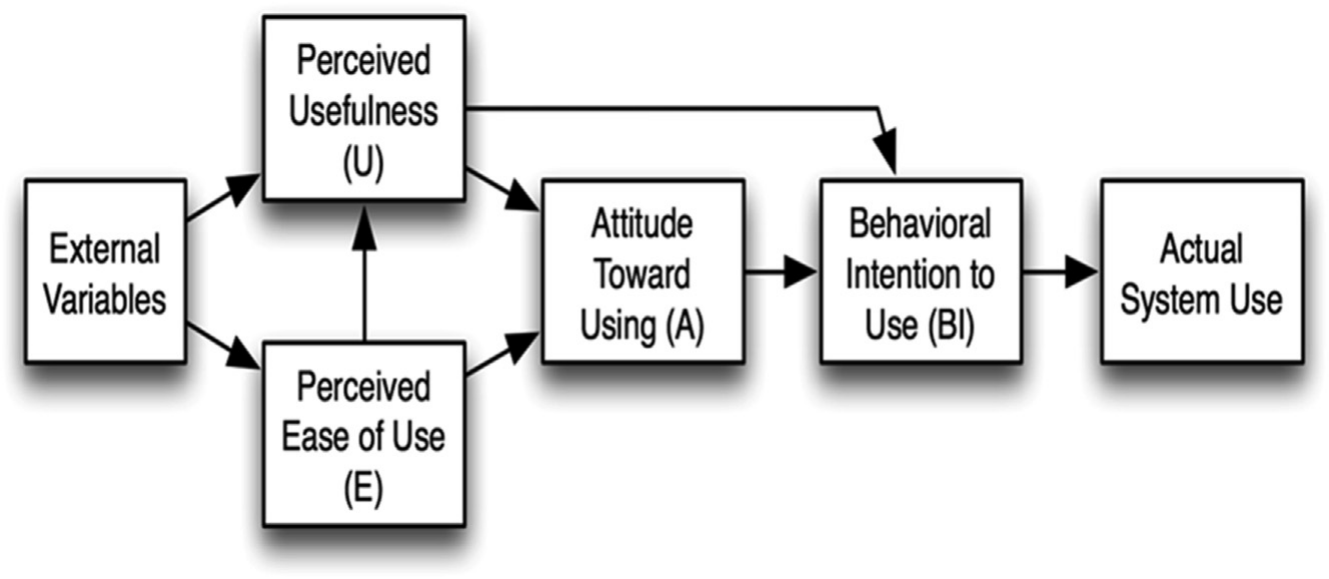
The original version of the Technology Acceptance Model.

This research uses the Technology Acceptance Model (TAM), as shown in Figure 1, as a leading and underpinning theory in the proposed research framework. TAM was developed by Davis [27], which is considered the most used theory in empirical research. This theory proposed by Davis [27], established on the Theory of Reasoned Action (TRA), has been described as a credible model for assisting the evaluation of various technology systems. It has been considered the critical model for knowing the predictors of human behaviour toward technology adoption [28]. TAM is the furthermost standard ground theory [29]. The study of Mokhtar, Katan [30] recommended further research on external variables influencing the perceived usefulness and perceived ease of use. Scherer, Siddiq [31] argued that it is insufficient to use the only TAM to explain all the relationships between technology systems and adoption behaviours. Meanwhile, TAM only consists of explanatory factors of perceived usefulness and ease of use [28]. However, the two main variables of TAM do not fully reflect factors that can encourage people's adoption of a technology system.

Perceived ease of use (PEOU) “refers to the degree to which the prospective user expects the target system to be free of effort” [32]. In this research, PEOU refers to the degree to which academicians believe that using LMS will be free of effort. If LMS is easy to use, offers benefits, and provides academic facilities to the lecturers, LMS will be more acceptable and utilised by lecturers, like accepting any particular information system [33].

Many studies revealed that PEOU had a significant direct influence on LMS usage. Ngai, Poon [34] proved the significance of the path linked from PEOU to LMS utilisation. The result of the study revealed that PEOU had a significant influence on LMS usage.

Perceived usefulness (PU) is “defined as the prospective user's subjective probability that using a specific application system will increase his or her job performance within an organisational context” [32]. In this study, PU refers to how academicians believe how much using LMS help them in their academic job.

Many studies related to LMS utilisation found PU to be the significant driver of actual usage among academicians [35, 36, 37]. For example, Fathema, Shannon [38] stated an extended TAM in the LMS context among the academicians. The result validated a significant influence of both PEOU and PU on the actual LMS.

Actual Use (AU) is defined by Davis [39] as an “individual's actual direct usage of a given system in the context of his or her job” [39]. In this study, the researchers refer to the lecturers' actual use of LMS as their consistency in using LMS tools such as content development, course delivery, assessment, and administrative tools for their teaching and learning process [40]. LMS use depends on how the system is considered to ease of use and usefulness and leads to academic achievements [41].

Related research [42] studied factors that affect university lecturers' use of different roles and capabilities of eLearning contexts. Mahdizadeh, Biemans [42] noted that university teachers’ perception of eLearning directly influences the actual use of the eLearning environment. Moreover, they found that PEOU and PU have a positive relationship with the actual use of eLearning.

## 4 Research model and hypotheses development

**Figure 2:**
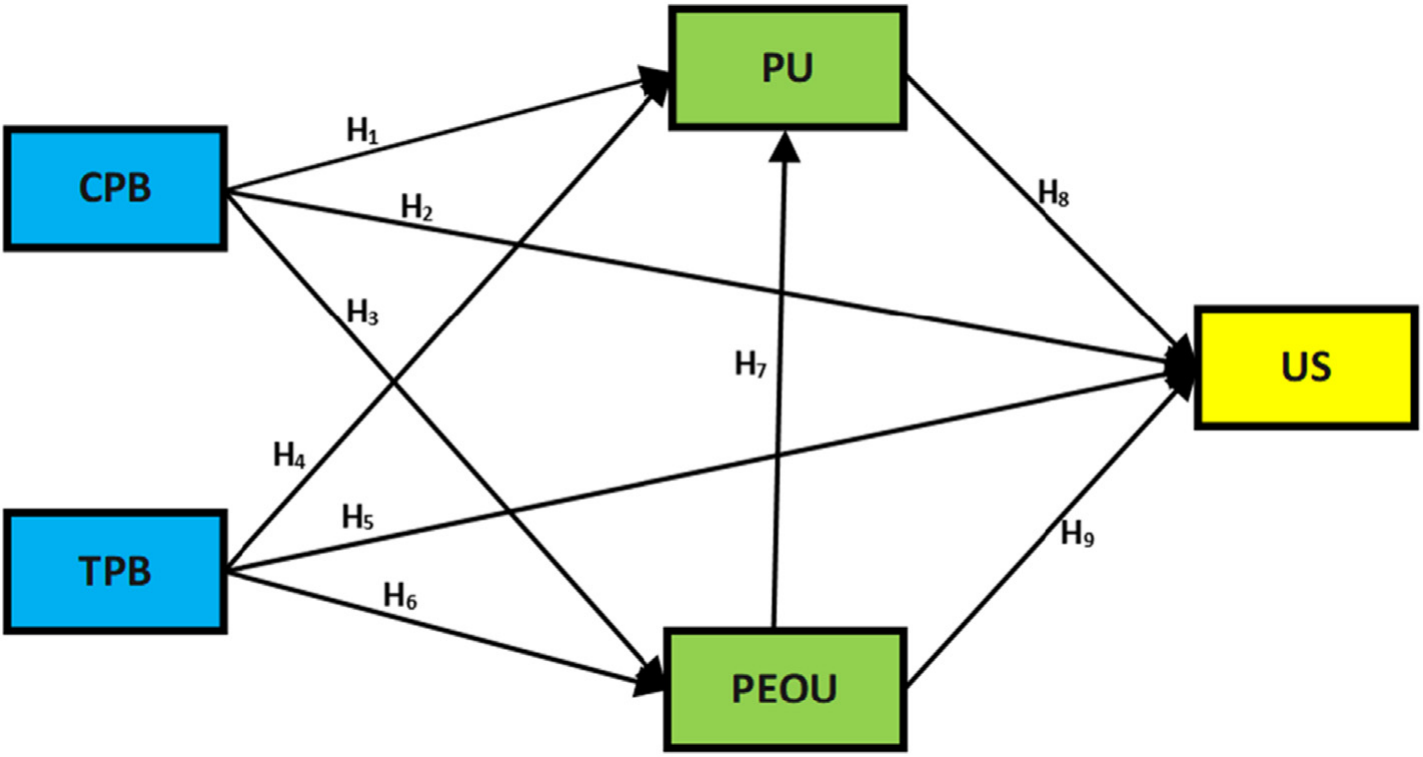
The proposed model is based on Technology Acceptance Model (Davies et al., 1989) (Note TPB: traditional pedagogical beliefs; CPB: constructivist pedagogical beliefs; PEOU: perceived ease of use; PU: perceived usefulness; US: Moodle LMS usage).

Regarding the research questions, the following figure presents a model of proposed relationships among constructs proposed to be tested in this research. The theoretical framework of this current study is based on the Technology Acceptance Model (TAM). Figure (2) indicates the theoretical framework of the current study.

Hence, this study hypothesises that traditional and constructivist pedagogical beliefs significantly affect Moodle LMS usage among academicians in the Iraqi Kurdistan Region. Previous studies have been extensively examined the relationship between pedagogical beliefs and their technology usage among teachers [14, 18, 19, 20]. It has been indicated that pedagogical beliefs have a significant role in choosing the technology system to align with their teaching strategy.

Ertmer, Ottenbreit-Leftwich [19], Judson [21] suggests that lecturers who hold student-centred beliefs tend to be more active technology users. Tondeur, Hermans [22] observe that lectures with constructivist beliefs are using technology. On the other side, Becker [23] argued that lectures that hold teacher-centred beliefs also tend to use technology in their classroom but might not use it in a more student-centred style.

Asiri [43] carried out research to address factors affecting the use of learning management systems in Saudi Arabian universities. The study's primary purpose was to develop a theoretical framework underlying research on factors that affect the utilisation of the Jusur Learning Management System (Jusur LMS) in Saudi Arabian public universities. One of the exciting findings of his study indicated that lecturers' pedagogical beliefs were found to play critical roles in LMS adoption and utilisation.

A study of faculty members from Greece found a relation between pedagogical beliefs and integrating LMS in teaching processes. They found that lecturers’ pedagogical beliefs are said to be a significant motivator for LMS and give many disciplines to faculty members [24].

Hence, this study hypothesises that traditional pedagogical beliefs and constructivist pedagogical beliefs significantly affect LMS usage among academicians in the Iraqi Kurdistan Region. As a result, the study hypothesized:

H1 There is a significant relationship between constructivist pedagogical beliefs and Perceived usefulness among academicians in the Iraqi Kurdistan Region.

H2 There is a significant relationship between constructivist pedagogical beliefs and Perceived ease of use among academicians in the Iraqi Kurdistan Region.

H3 There is a significant relationship between constructivist pedagogical beliefs and Moodle LMS usage among academicians in the Iraqi Kurdistan Region.

H4 There is a significant relationship between traditional pedagogical beliefs and Perceived usefulness among academicians in the Iraqi Kurdistan Region.

H5 There is a significant relationship between traditional pedagogical beliefs and Perceived ease of use among academicians in the Iraqi Kurdistan Region.

H6 There is a significant relationship between traditional pedagogical beliefs and Moodle LMS usage among academicians in the Iraqi Kurdistan Region

The leading dimension stares at the emotional observation of the person regarding system performance and individual trust that LMS usage will have future job benefits [44]. This dimension, created on the TAM, contains perceived usefulness (PU), perceived ease of use (PEOU), and actual use. The study proposed that PU have a positive direct impact on the actual use as lecturers will be gland to use LMS if they believe that the system is useful.

Many researchers provided evidence that PEOU has a direct influence on actual use. Perceived usefulness from the lecturer's perspectives possibly will affect their action on the road to adopting LMS [45]. It has been stated that users may reject learning new technology systems because of the difficulty of the new system [46]. As a result, PEOU in LMS might influence the intention of lecturers in KRI to accept LMS. Hence, the following hypothesises formulated:

H7 There is a significant relationship between Perceived ease of use and Perceived usefulness among academicians in the Iraqi Kurdistan Region.

H8 There is a significant relationship between Perceived usefulness and Moodle LMS usage among academicians in the Iraqi Kurdistan Region

H9 There is a significant relationship between Perceived ease of use and Moodle LMS usage among academicians in the Iraqi Kurdistan Region

## 5 Methodology

### 5.1 Participants

Since the target population in this research is the academic staff of the private and public universities in the Kurdistan Region, the sample for this study will be taken from universities listed under the Ministry of higher education of KRG. University lecturers (N = 393) from eight universities in the Kurdistan Region of Iraq participated in the current study. As shown in Table 1, they included 217 males and 176 females. Nearly half of the participants (N = 190, or %40.46) revealed that they were aged between 36 and 45 years. Almost all of them had a master's degree (N = 211) or a doctoral degree (N = 153). Only 25 of the participants' academic ranking were professors due to having a limited number of professors in the Iraq universities.

**Table 1:** Participant descriptive analysis.

Gender	Frequency	Percent
Male	217	55.22
Female	176	44.78
**Age**	**Frequency**	**Percent**
25–30 years	22	5.60
31–35 years	56	14.25
36–40 years	103	26.21
41–45 years	87	22.14
46–50 years	51	12.98
51–55 years	32	8.14
56–60 years	22	5.60
Over 60 years	20	5.09
**Education**	**Frequency**	**Percent**
High Diploma	29	7.38
Master	211	53.69
PhD	153	38.93
**Academic Ranking**	**Frequency**	**Percent**
Assistance Lecturer	151	38.42
Lecturer	149	37.91
Assistance Professor	68	17.30
Professor	25	6.36

### 5.2 Measures

This present study used a survey as a research method to collect data from academicians. The researchers found that the survey is appropriate for collecting data. The data will be collected through the hand by hand distribution of the survey questionnaire to the academicians in public and private universities in KRI. The questionnaire will comprised of close-ended questions using English and will be translated into the Kurdish language. The questionnaire is divided into three sections. The first section is about the demographical information of the academicians. In contrast, the second section is on the LMS usage using the following items, as shown in Table 2.

**Table 2:** Construct measurement.

Construct	Item No.	Items
Constructivist Pedagogical Beliefs	CPB1	“Learning means students have full opportunities to explore, discuss and express their ideas.”
	CPB2	“Every student is unique or special and deserves an education tailored to his or her particular needs.”
	CPB3	“It is important that a teacher understands the feelings of the students.”
	CPB4	“Good teachers always encourage students to think for answers themselves.
	CPB5	“In good classrooms, there is a democratic and free atmosphere which stimulates students to think and interact.”
Traditional Pedagogical beliefs	TPB1	“During the class, it is important to keep students confined to the textbooks and the desks.”
	TPB2	“Learning to teach simply means practising the ideas from lecturers without questioning them.”
	TPB3	“Teaching is simply telling, presenting, or explaining the subject matter.”
	TPB4	“Good teaching occurs when there is mostly teacher talk in the classroom.”
	TPB5	“Teaching is to provide students with accurate and complete knowledge rather than encourage them to discover it.”
Perceived Usefulness	PU1	“Using LMS for work enables me to accomplish tasks more quickly.”
	PU2	“Using LMS for work improve my job performance.”
	PU3	“Using LMS for work increases my job productivity.”
	PU4	“Using LMS for work enhances my effectiveness.”
	PU5	“LMS for work is useful in my job.”
Perceived Ease of Use	PEOU1	“Learning how to use LMS is easy.”
	PEOU2	“My interaction with LMS is clear and understandable.”
	PEOU3	“I find LMS to be very flexible.”
	PEOU4	“I find it easy to get LMS to do the work I want it to do.”
	PEOU5	“Overall, I find that LMS is easy to use”
Moodle LMS usage	US1	Currently, I use LMS in my teaching and learning process
	US2	I use LMS more than any other educational technologies?
	US3	I use LMS to upload course materials?
	US4	I use assessment tools (quiz or test) inside LMS?
	US5	I use communicating tools (discussion) inside LMS?

In contrast, the final section consists of the items related to the pedagogical beliefs constructs used in this study. Lectures have been asked about their technology acceptance in three constructs: PEOU (five items), PU (five items) and Moodle LMS usage (five items). The items were based on TAM designed by Davis, Bagozzi [32]. The remaining items related to the lecture's pedagogical beliefs, with five covering constructivist pedagogical beliefs and the other five coving traditional pedagogical beliefs. Regarding measuring pedagogical beliefs, this study focuses on how academicians' essential pedagogical beliefs, such as constructivist and traditional influence on LMS's usefulness and ease of use, and their actual use, to measure the effectiveness of the TAM in the academic setting in Iraqi Kurdistan Region. This study adopts the instrument from the study of Teo, Chai [47], Liu, Lin [48] and Chan and Elliott [49].

### 5.3 Data collection procedure and analysis

The data collection process will start by administering the questionnaires directly or mail to the respondents. Secondly, the researcher can directly walk into the identified clusters and administer the questionnaire with the permission of the university's administration. The data were collected from university lecturers who were lecturing and familiar with LMS at the universities under the rule of MOHSR. The study and its purpose were clarified to the lecturers, and they were asked whether they were willing to participate in the study. Before completing the questionnaire, they were asked to read and sign the consent letter, which ensured them that their participation was voluntary and that their responses were confidential. A consent letter was obtained from all participants, and they were requested to read the instruction and carry out the survey accordingly. They were persuaded to ask questions about any vague items in the questionnaire. Consequently, ethical considerations were carefully observed during data collection. The approving ethical committee for this research was called Kurdistan National Research Council Ethical Committee (KNRCEC).

As this study is not based on a developing construct scale, this will use a developed questionnaire from previous studies. In doing so, factors analysis and reliability tests are required to determine the likelihood of effectiveness of the questionnaire. Exploratory and confirmatory factor analysis are required to check for the validity of a new measure and adopted measures, respectively [50]. Using statistical tools is often appropriate for multivariate data analysis through Partial Least Square (PLS-SEM), also known as PLS path Modelling. PLS-SEM helps develop theories in an exploratory study by concentrating on the adjustments in the dependent variable when examining a model. According to Hair, Hult [50], an exploratory instrument is used when a researcher examines which independent variables have a high potential to predict the best outcome on a dependent variable or when the research goal is to determine a statistically significant predictor confirmatory indicator.

## 6 Results

### 6.1 Assessment of data normality

It is necessary to check the data normality before doing any statistical analysis because the researchers choose the appropriate test based on the data normality [51]. Micceri [51] also commented that a significant amount of literature had been keen on the need for normal distribution in using analytical tools and techniques in the analysis. This study used skewness and kurtosis to identify the normality of the data distributions and presented the mean and standard deviation as shown in the following Table 3.

**Table 3:** The mean and standard deviation with skewness and kurtosis for the Items.

Items	Mean	Std. Deviation	Skewness		Kurtosis	
	Statistic	Statistic	Statistic	Std. Error	Statistic	Std. Error
CPB1	3.519	1.116	-0.530	0.123	-0.316	0.246
CPB2	3.499	1.010	-0.541	0.123	-0.135	0.246
CPB3	3.819	1.081	-0.670	0.123	-0.364	0.246
CPB4	3.860	1.092	-0.854	0.123	0.143	0.246
CPB5	3.690	1.079	-0.632	0.123	-0.111	0.246
TPB1	3.254	1.107	-0.256	0.123	-0.646	0.246
TPB2	2.893	1.149	-0.074	0.123	-0.830	0.246
TPB3	3.239	1.138	-0.323	0.123	-0.773	0.246
TPB4	3.145	1.166	-0.139	0.123	-0.926	0.246
TPB5	3.071	1.225	-0.195	0.123	-0.923	0.246
PU1	3.440	1.162	-0.541	0.123	-0.453	0.246
PU2	3.461	1.044	-0.388	0.123	-0.406	0.246
PU3	3.463	1.106	-0.344	0.123	-0.635	0.246
PU4	3.458	1.085	-0.433	0.123	-0.412	0.246
PU5	3.565	1.070	-0.497	0.123	-0.328	0.246
PEU1	3.565	1.146	-0.544	0.123	-0.481	0.246
PEU2	3.570	1.084	-0.677	0.123	-0.079	0.246
PEU3	3.560	1.068	-0.504	0.123	-0.369	0.246
PEU4	3.547	1.044	-0.551	0.123	-0.142	0.246
PEU5	3.573	1.114	-0.545	0.123	-0.414	0.246
LMS Usage1	3.664	1.134	-0.633	0.123	-0.320	0.246
LMS Usage2	3.501	1.114	-0.360	0.123	-0.701	0.246
LMS Usage3	3.598	1.114	-0.537	0.123	-0.463	0.246
LMS Usage4	3.254	1.179	-0.279	0.123	-0.722	0.246
LMS Usage5	3.140	1.291	-0.270	0.123	-0.984	0.246

### 6.2 Assessment of the measurement model

In the measurement model evaluation step, the composite reliability and Cronbach's alpha are examined for assessing construct reliability. Secondly, convergent validity and discriminant validity were also checked to observe that the items have adequate capacity to converse towards their construct. In the case of discriminant validity, we observe that all the constructs are distinctive and separate.

### 6.3 Construct validity and reliability

Checking the item level reliability is the first criteria for examining the internal consistency of the items by measuring the items are internally consistent. Mainly, the underlying constructs explain the items variance, which signifies item reliability. Chin [52] recommends that the latent variable proves the standardised factor loadings which required more than or equal to 0.50 or 50% [53]. recommend that the factor loadings need to be higher than 0.70. However, Churchill and Gilbert [54] suggests that the outer loadings should not be less than 0.4. Table 4 shows the result of the measurement model analysis, which manifests that the outer loadings are between 0.751 to 0.893, above the minimum threshold criterion [52, 53, 54, 55].

**Table 4:** Internal consistency and convergence validity results. Notes: CR: Composite Reliability; AVE: Average Variance Extracted; CA: Cronbach's Alpha.

Constructs	Items	FL	CA	CR	AVE
Constructivist pedagogical beliefs	CPB1	0.831	0.879	0.912	0.673
	CPB2	0.799			
	CPB3	0.834			
	CPB4	0.844			
	CPB5	0.795			
Perceived ease of use	PEOU1	0.826	0.901	0.926	0.716
	PEOU2	0.867			
	PEOU3	0.847			
	PEOU4	0.831			
	PEOU5	0.858			
Perceived usefulness	PU1	0.825	0.893	0.921	0.700
	PU2	0.846			
	PU3	0.840			
	PU4	0.832			
	PU5	0.840			
Traditional pedagogical beliefs	TPB1	0.797	0.880	0.913	0.677
	TPB2	0.826			
	TPB3	0.847			
	TPB4	0.812			
	TPB5	0.829			
LMS usage	US1	0.797	0.851	0.893	0.626
	US2	0.810			
	US3	0.829			
	US4	0.769			
	US5	0.748			

**Figure 3:**
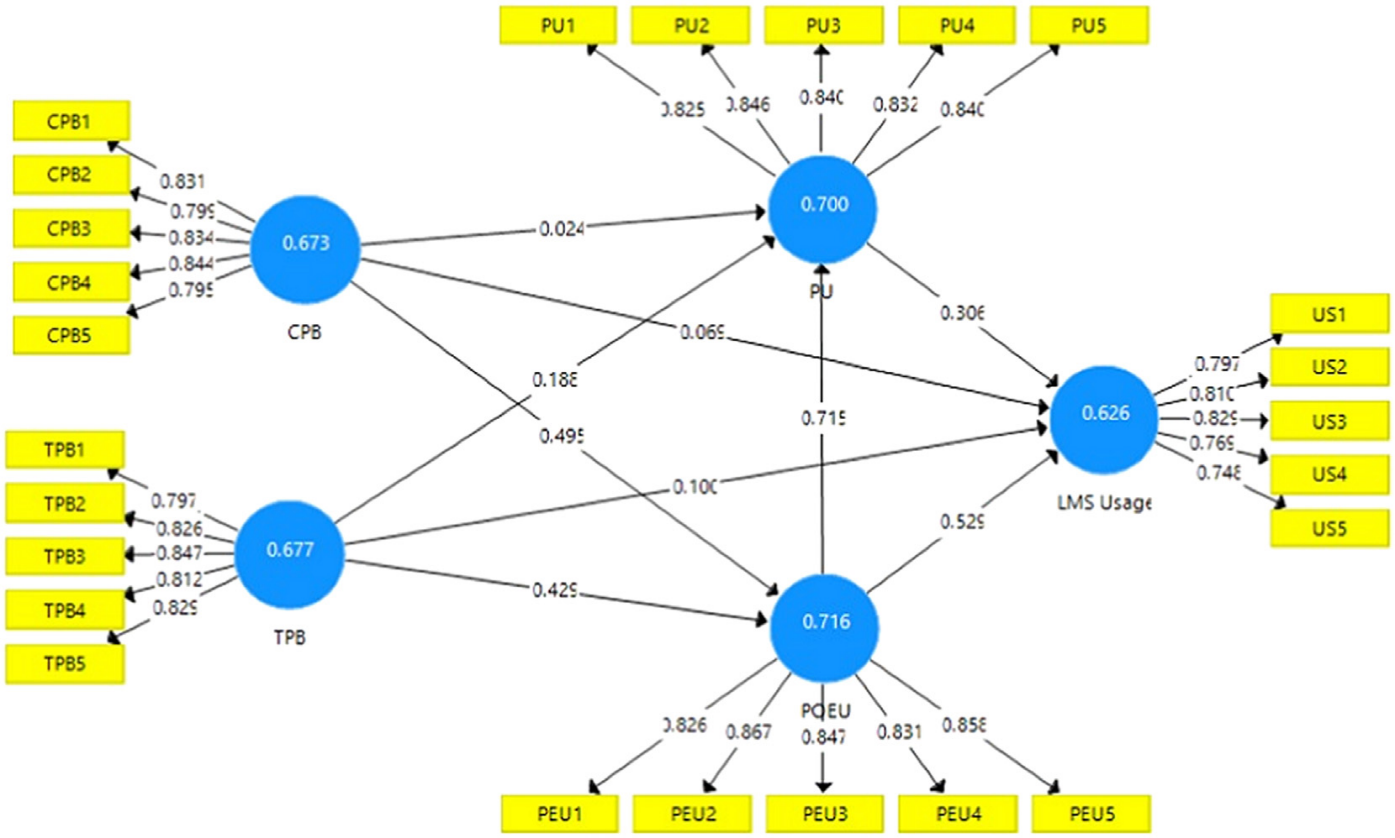
Presented the factor loadings and AVE of all constructs through PLS-Algorithm.

In the current research, convergent validity is tested by using the universally established method “Average Variance Extracted” (AVE) [53, 55, 56]. Table 4 shows that the AVE for each latent variable is more significant than the suggested value of 0.5 (50%), indicating that each construct could clarify more than half of the variance to its measuring items on average [57]. The factor loading is shown in Table 4 and Figure 3.

### 6.4 Measurement of discriminant validity

Discriminant validity determines the difference between one construct from other constructs. Table 5 below describes the abstract for discriminant validity. Regarding the table, the discriminant validity is reached when a bold diagonal value is higher than its row and column values. It has many approaches for determining discriminant validity like hetero-trait and mono-trait HTMT, Cross Loading, and Fornell Larcker. The first criterion for confirming the discriminant validity is Fornell Larcker. As per the Fornell Larcker criteria, the square root value of the AVE of one construct must be higher than the inter-correlations between the constructs. As presented in Figure 3, the square roots of the AVE values of all variables are higher than their respected inter-correlation values.

**Table 5:** Discriminant Validity – Fornell and Lacker Criterion. The off-diagonal values are the correlations between latent variables, and the diagonal is the square root of AVE.

	CPB	POEU	PU	TPB	US
CPB	0.821				
POEU	0.576	0.846			
PU	0.472	0.828	0.837		
TPB	0.191	0.524	0.567	0.823	
US	0.538	0.875	0.834	0.564	0.791

### 6.5 Direct effect (path coefficient) analysis

The path coefficient in Smart-PLS is similar to the standardised β in the multiple regression analysis. Table 6 shows the path coefficient evaluation outcome where all the nine hypotheses were supported. The supported hypotheses are significant at least at the level of 0.05, have assumed sign directions and consist of a path coefficient value (β) ranging from 0.051 to 0.417.

**Table 6:** Path coefficient result, Significant: p < 0.05

Hypotheses	OS/Beta	LL	UL	T	P	Decision
CPB- > POEU	0.567	0.500	0.628	14.547	0.000	Significant
CPB - > PU	0.140	0.064	0.216	2.994	0.001	Significant
CPB - > US	0.122	0.053	0.187	3.018	0.001	Significant
POEU - > PU	0.623	0.546	0.692	13.983	0.000	Significant
POEU - > US	0.572	0.486	0.657	11.038	0.000	Significant
PU - > US	0.155	0.063	0.246	2.811	0.002	Significant
TPB- > POEU	0.091	0.031	0.163	2.285	0.011	Significant
TPB - > PU	0.091	0.034	0.154	2.463	0.007	Significant
TPB - > US	0.056	0.007	0.105	1.907	0.028	Significant

The highly significant path (p = 0.000) was found between PEOU and PU (β = 0.623), the second highly significant path (p = 0.000) was between PEOU and US (β = 0.572), and the third highly significant path was between CPB and POEU as the (p = 0.000) and the β = 0.567. The fourth highly significant path was between PU and US as the p = 0.002 and the β = 0.155. The fifth highly significant path was between CPB AND PU as the p = 0.001 and the β = 0.140. The sixth highly significant path was between CPB and US as the p = 0.001 and the β = 0.122. Both of the paths (TPB - > POEU and TPB - > PU) have the same significant path β = 0.091. Furthermore, the least significant path (p = 0.028) was between TPB and US (β = 0.056).

### 6.6 Findings

This study showed that constructivist pedagogical beliefs (CPB) have a significant relationship with Moodle LMS usage, with a standard beta value of 0.122 and a t-value of 3.018. It means that those lecturers who have constructivist pedagogical beliefs influenced Moodle LMS usage. This result collaborates with the study of Liu, Lin [48] and Becker [23], finding the significant result of educators' constructivist beliefs with computer use. CPB does have a direct predictive effect on PU and PEOU, thus confirming that the methodology used by the lecturers influences the usefulness and ease of use of LMSs. It can be interpreted that lecturers who have constructivist approaches automatically tend to adapt to new technologies with the perceived need for some valuable and easy to use. Therefore, lecturers with constructivist pedagogical beliefs are more aware of LMS platforms’ advantages in supporting constructivist learning activities.

TPB was found to significantly impact lecturers’ LMS usage, with a standard beta of 0.056 and t-value of 1.907. This result is consistent with previous studies [58], which reported that TPB was significantly correlated with beliefs in technology integration. While the result of this study is confounding with Tondeur, Hermans [22] that lecturers who hold traditional pedagogical beliefs do not perceive to use of technology in their teaching. The result showed that TPB has a significant influence on PU and PEU. This result is consistent with the current study results of [59] and from prior studies [48, 58], which reported that the relationship between TPB with PU and PEU is not significant.

This study finds exciting results in the technology acceptance model for Moodle LMS usage. For instance, most past studies that use TAM show the most vital relationship between perceived usefulness, perceived ease of use, and actual use. A few studies also show the most vital relationship between mentioned TAM constructs. Therefore, the finding of this research goes with the famous school of thought, which is the most vital relationship between perceived usefulness, ease of use and Moodle LMS usage.

## 7 Discussion

It can be noted from the result of this study that both CBP and TPB have a significant relationship directly with LMS usage and so on. Both of them have a significant relationship with PU and PEU. This confounding result can be explained in three reasonable ways; The first is that most participant lecturers hold both pedagogical beliefs [59]. Might be participant lecturers utilise a “blended” pedagogical approach (using traditional and constructivist pedagogical beliefs), allowing them to merge differences between their student-centred beliefs and their teacher-centred practices. The second is that, for some lectures, those who agree with two pedagogical beliefs might be the dual influence from traditional Arabic culture learning and the push on educational reform efforts from the Ministry of Higher Education in the Kurdistan Region. These lecturers are more tolerant of conflicting lecturing philosophies and are more flexible in applying different methodologies in their teaching for different reasons. It may be that lecturers with dual high pedagogical beliefs can better select and apply technologies in different teaching contexts (both as a tool or for higher learning). The third reason is that the data collected during the pandemic forced all the lecturers to use LMS because of the lockdown. This makes a different type of pedagogical belief holders use LMS.

This study has several theoretical and practical contributions to online learning literature. First, it examined constructivist and traditional pedagogical beliefs as factors influencing LMS usage. Past studies have recommended exploring factors that influence LMS usage. The current research presents significant perceptions of how constructivist and traditional pedagogical beliefs affect LMS usage. Unexpectedly, the results show that constructivist and traditional pedagogical beliefs have the same impacts on LMS usage during the COVID-19 pandemic.

Moreover, the research has extended previous literature of the TAM model in examining LMS usage. As an outcome of the study, constructivist and traditional pedagogical beliefs, extended as external factors into the original TAM model as external factors, play a critical role in explaining how actual technology uses are deployed through perceived ease of use and perceived usefulness within the online learning environment.

Nevertheless, prior research has shown the impacts of pedagogical beliefs on LMS adoption independently through various contexts. However, both pedagogical factors determined in this study are rarely integrated into a study to examine the actual use of educational technology within the online platform. The current research improves the understanding of the combined effects of constructivist and traditional pedagogical beliefs on LMS usage.

Moreover, the study reveals that perceived usefulness and perceived ease of use play critical roles in explaining how LMS actual use is affected in the current theoretical model. First, they have a direct impact on actual use. It means that lecturers’ perception and experience toward the LMS platforms will affect their actual use of technology. Therefore, university leaders must make their LMS a helpful platform and ease of use. For example, the LMS platform should include information and characteristics that enhance the teaching procedures and are much easier to use during the teaching period.

For the policymakers in KRG, the findings of this study demonstrate that they should be serious about announcing a new policy to involve all the academicians with different pedagogical beliefs to use eLearning within teaching practice.

The findings of this research also have some implications for university lecturers and policymakers. The lecturers who use LMS revealed the significance of pedagogical beliefs in technology integration development. Thus, teacher educators should understand lecturers' pedagogical beliefs and perceptions in course design. To shape academic staff's pedagogical beliefs in constructivism, they would train their students in more constructivist ways and model how to employ constructivist teaching strategies and activities.

Finally, constructivist and traditional pedagogical beliefs indirectly affect online LMS usage through perceived ease of use and usefulness. Thus, the online industries that develop the LMS platforms for educational institutions must understand the nature of these factors' relationships before expanding the LMS platforms. They are advised to improve LMS platforms’ performance.

## 8 Conclusion

The study concludes that the COVID-19 pandemic impacts lecturers to use LMS even though they hold deference pedagogical beliefs. The significance of this study is incorporating pedagogical beliefs into the Technology Acceptance Model for university lecturers in the Kurdistan Region. This paper indicates that TAM could effectively explain lecturers’ LMS usage in broader and more diverse contexts. The study reveals that university lecturers were more constructivist-oriented and positively influenced by the LMS actual usage and traditional pedagogical beliefs found that it has a significant positive relationship with perceived ease of use, usefulness, and LMS usage. One of the limitations that should be considered is that this study does not include other significant constructs such as facilitating conditions, social influence, and government support. One more limitation is that survey collected the data during the COVID-19 pandemic. It was better to use a mixed-method combing some qualitative methods.

Moreover, this study focused on only one LMS platform, namely Moodle LMS. It is better to do a study on all the LMS types. Last and never least, another limitation is that this study did not include any articles published in languages other than English. Such an inability to access some publications may lead to various biases.

To ensure the correct spread and LMS usage in the future, utilising LMS during the teaching process may no longer be elective but must be required so that classes will not be interrupted when other pandemics may affect the lesson's delivery. This current pandemic and predicting future crises make lecturers realise how important it is to have LMS that can be used when future disruptions are expected. Having a straightforward and established LMS may be excellent support when shifting from face-to-face classes to online. Therefore, in times of pandemics and emergency crises that affect classroom teaching, the use of LMS should be considered pedagogically.

## Declarations

### Author contribution statement

Twana Tahseen Sulaiman: Conceived and designed the experiments; Performed the experiments; Analyzed and interpreted the data; Contributed reagents, materials, analysis tools or data; Wrote the paper.

Anuar Shah Bali Mahomed: Conceived and designed the experiments; Contributed reagents, materials, analysis tools or data.

Azmawani Abd Rahman: Performed the experiments.

Mazlan Hassan: Analyzed and interpreted the data.

### Funding statement

This research did not receive any specific grant from funding agencies in the public, commercial, or not-for-profit sectors.

### Data availability statement

Data included in article/supplementary material/referenced in article.

### Declaration of interest’s statement

The authors declare no conflict of interest.

### Additional information

No additional information is available for this paper.
